# "The Hospital" Nursing Mirror

**Published:** 1899-09-23

**Authors:** 


### "The Hospital" Nursing Mirror.

Being the Nursing Section of "The Hospital."

[Contributions for this Section of "The Hospital" should be addressed to the Editor, The Hospital, 28 & 29, Southampton Street, Strand, London, W.C., and should have the word "Nursing" plainly written in left-hand top corner of the envelope.]

### Notes on News from the Nursing World.

### NURSING IN THE TRANSVAAL.

At any moment there may be a considerable demand for nurses to proceed to the Transvaal. We have already received a number of inquiries from nurses wishing to volunteer for the work, and, in reply to questions, have pointed out that if further nursing than that guaranteed by the Army Nursing Service is required, the Army Nursing Service Reserve will be called out. Full details of the qualifications required by nurses anxious to enrol themselves in the latter will be found in a Chat with Lieut.-Colonel Gubbins, the secretary, which appeared in these pages on May 13th last. In time of war, it may be repeated, the Nursing Reserve is under the control of the War Office; and one of the qualifications that gives preference in selection is the ability to speak and write one or more foreign languages.

### NUNS AS NURSES.

Elsewhere a contributor raises the interesting question whether nursing by lay nurses as a profession, or by spiritual sisters as a devotion is the better. Our contributor, who deals only with the point as it affects French hospitals, puts the case on both sides fairly enough so far as it is pursued. In Ireland it is still exciting particular interest just now, and we see that Lord Stopford and Captain Richards have resigned their seats as guardians of the Gorey Union because it has been decided by the board to employ three nuns as nurses. According to the *Irish Independent* Captain Richards objected on the ground that the vows of nuns preclude them from assisting at surgical operations on male patients.

### NURSING IN SCOTTISH PRISONS.

The committee appointed to investigate the provision for the nursing and accommodation of sick prisoners in Scottish prisons will shortly hold a preliminary meeting. They expect to get to work in about six weeks, but we learn that the evidence is likely to be kept private until it is published with the report. Without for a moment suggesting that there is any danger of the suppression of unpleasant facts, we believe that the admission of representatives of the press to the meetings of the committee would be an advantage, and would distinctly tend to inspire the public with confidence as to the outcome of the inquiry.

### OUR CONVALESCENT FUND.

The summer is nearly over, and holiday makers are returning to work. Now is, therefore, the time for convalescents, too poor to pay the season's prices, to find rest and renewed strength by the sea or in the country at reasonable charges. Though the heat has been succeeded by a cooler atmosphere there are probably many beautiful autumn days to come, and the sharpness in the air is the best of tonics to those strong enough to stand it. Therefore, we are sure our kind readers will forgive us for reminding them that stray half-crowns or smaller contributions, as well as sums of greater value, are necessary to keep our convalescent fund alive. For the sake of new subscribers it is worth repeating that this fund is supported by contributions from readers of The Hospital, principally nurses, to send other and poorer nurses recovering from illness to a convalescent home. The fund generally manages to give three weeks' change and to contribute 5s. towards travelling expenses. But, as efforts are made to send convalescents to places which seem most appropriate, allowance must be made for occasionally increased expense. Each case is recommended by the applicant's medical attendant and by a householder, a necessary precaution to ensure that none but nurses benefit by a nurses' fund.

### THE DEMAND FOR INSTRUCTIONS IN SICK NURSING.

A striking proof of the increasing recognition of the importance of nursing is afforded by the replies sent to a circular issued under the auspices of the Technical Sub-Committee of the Aberdeen County Committee on Secondary Education. The sub-committee invited a number of parishes to state their views as to the subjects on which they would like to have instruction given by experts. The result was that six parishes selected cooking, three poultry keeping, and sixteen sick nursing. In these circumstances the sub-committee have very naturally resolved to proceed at once with the appointment of an instructress for sick nursing.

### THE PROBATIONER SYSTEM IN WORKHOUSE INFIRMARIES.

In East Anglia two Boards of Guardians have decided to dispense with probationers. Apparently, the real reason is that they cannot get them. But at a meeting of the Lowestoft Board a very foolish objection was raised by the master of the workhouse. He said that "the old people in the infirmary did not like young women to look after them," and that "the nurses should not be under 30 years of age." Sympathy with the victims of the "too-old-at-forty" cry does not prevent us from regretting any unreasonable attempt in the other direction. If men between 60 and 70 are often in their prime, and all efforts to fix an age limit to human endeavour must necessarily fail, a probationer between 20 and 30, if she is competent to nurse at all, is quite able to attend to the requirements of old people in an infirmary.

### PROGRESS IN HONG KONG.

On condition that the Chinese community in Hong Kong will subscribe sufficient funds in 18 months to erect an extension of Tung Wa Hospital, the Colonial Secretary, at the instance of the Governor, has granted a site. In this building it is proposed to set aside a special department for midwifery, which is urgently needed, owing to the high mortality amongst infants and mothers. The extension scheme also includes a permanent building for the treatment of plague patients, the present mat sheds being considered unsafe in case of fire or typhoon. Nurses do not object to take ordinary risks, but there should be absolute protection against the elements.

### THE DRAMA AND THE DANCE.

From Worcestershire comes a report of a representation of "As You Like It" in the grounds of Lower Court, Kemerton, on behalf of the Rural Nursing Association. About two hundred people were present at the performance, which, thanks to the kindness of Mr. and Mrs. Phillips, was carried out under such delightful conditions, and a sum of over £20 was realised for the charity. In quite another part of the country, at Talsarnau, North Wales, a subscription dance was given at the Board School for the benefit of the Harlech District Nursing Association. Dancing was kept up with spirit from eight to one, and, after payment of all expenses, £15 was handed over to the fund.

### THE IMPROVEMENTS AT ST. GEORGE'S HOSPITAL.

It will still be some time before the improvements and alterations at St. George's Hospital are completed. Meanwhile, the hospital is full to overflowing, and the staff has, therefore, to be kept up to its ordinary strength. Of course, this does not mean that the nurses have not had any holidays, for vacations must be arranged for in the routine of the work in every institution. But the full beds make it impossible to dispense with the services of a large proportion of the staff at a time. Fortunately, the authorities have accommodation for about a dozen nurses at the nurses' home, and this is used whilst the hospital quarters are being reconstructed by degrees. Our readers may remember that two of St. George's nurses went more than a year ago to the municipal hospital at Rangoon, to be in readiness in case the plague made its appearance. Their friends at the hospital continue to receive very satisfactory letters from them.

### IMPROVED ACCOMMODATION FOR AMERICAN NURSES.

A new home for the nurses of the City Hospital, Rochester, New York, will be ready for dedication in October. It will accommodate 60 nurses, and the cost of the building, which is on a very handsome scale, is to be nearly 30,000 dollars. The extension of the Women's Hospital at Saginaw, Michigan, which is about to be commenced, will include a large dormitory on the third floor for nurses. A new nurses' dormitory, three stories high, is to be erected for the nurses of Harper Hospital, at Detroit; and the nurses of the Union Protestant Infirmary, Baltimore, who now live in the infirmary, are shortly to have a separate home provided for them.

### NURSES AND THE NEW PRESIDENT OF THE OPHTHALMOLOGICAL SOCIETY.

The appointment of Mr. Anderson Critchett to the post of President of the Ophthalmological Society seems to have given particular pleasure to nurses. One of these writes in most enthusiastic terms about him, and mentions his kindness to those in need. "For example," she says, "a nurse I know, before she entered the profession, consulted him annually for several years. He happened to hear that she had met with money troubles, and the next time she went to him he said, 'Except you are willing to come and see me as a friend, without a fee, I will not see you at all.' This is only one instance; but such delicate benevolence does one good in this world of rush and struggle."

### HOSPITAL REFORM ABROAD.

Owing to a misunderstanding having arisen with reference to the note "Hospital Reform Abroad," in our issue of August 5th, our correspondent wishes to point out that the article "was written purely from a *nurse's* point of view, showing the state of things at the temporary hospital, where the work was taken up nearly a year ago, before the completion of the present building; not as a criticism on the present hospital and its management.

### ST. STEPHEN'S HOSPITAL, DELHI.

We learn from a report of the St. Stephen's Hospital, Delhi, how different work and life are there from the same work and life in England. In some ways they are more interesting, in others more disheartening. There is greater interest because the work amongst the poor native women means the opening of a new life and a new world for them. On the other hand, it is disheartening because, after a successful operation, the friends of the patients will often insist on moving them long before they are fit to be moved. The report says: "The bright wards, the cleanliness, above all the happiness of our children, and, indeed, generally speaking, of everybody, must appeal to the patients. These are from every caste—Hindu, Mohammedan, and Christian."

### A PATIENT'S JOKE.

"One night, just before going off duty," writes a nurse, "I fancied I spotted a temperature in old Marne, a pet patient of mine. I popped the thermometer under his arm and went to report to sister; she sent me straight off, promising to attend to Marne's temperature. The next morning when I entered my ward my attention was arrested by Marne's peculiar expression of face and exceedingly stiff attitude; walking to him I inquired the reason. "Why, Miss," he replied in an aggrieved tone, "here have I been awaiting for you all night to take this blessed glass from under my arm." "You old silly," I was beginning to say, when a laugh went round the ward. It seemed that sister had forgotten all about him in the press of an accident coming in, and the old rogue had carefully put the thermometer in his locker for the night, replaced it when he woke, and then had his little joke ready for me in the morning."

### SHORT ITEMS.

The respectable sum of £30 was realised by the demonstration on behalf of the North London Nursing Association at Holloway on Sunday week.—The street carnival at Gainsborough on behalf of the nursing association, which is now a regular annual event, was not only successful as a show, but yielded between £50 and £60.—At a meeting held last week by the Mutford and Tothingland Guardians, two probationers sent in their resignations. The board decided that the workhouse was not a suitable training place, and it was agreed that in future they would dispense with probationers.—An interesting and graphic "Sketch from Life" of a nurse of the ancient school appeared in the *Westminster Gazette* on Tuesday.

### The Nursing of Cases of Hernia.

By W. McAdam Eccles, M.S.Lond., F.R.C.S.Eng., Assistant Surgeon West London Hospital.

### I.

A hernia is the protrusion of any of the abdominal organs through some aperture in the abdominal wall. There are two classes of cases of hernia which may require to be submitted to operation, and it is intended in two lectures to review the duties which a nurse may be called upon to perform under such conditions. Operations upon hernia are of the nature of true abdominal operations, since in almost every instance the peritoneal cavity is opened. Thus the greater part of that which is stated here will apply with equal force to any operation of a similar kind. The two circumstances in which a hernia may need to be treated by operative measures differ considerably. In the one, it is the relief of the strangulation of imprisoned bowel that the surgeon has primarily before him, while in the other it is the cure of the protrusion which is being aimed at by the operator. The first operation is one of emergency, the second one of expediency; the one undertaken to save life, the other to rid the patient of an annoyance and possible danger.

It is not the purpose of these lectures to enter upon the surgical aspect of hernia in any detail, but should a nurse who reads them be not sufficiently familiar with the condition, it would be well for her to consult a text-book on surgery for a clearer idea of the subject. A strangulated hernia is a surgical emergency, for it is an intestinal obstruction, and its treatment brooks no delay if it is to be successful. In many, perhaps the majority, of the cases of strangulated hernia, the best treatment to relieve the imprisoned bowel of the constricting force is the operation of herniotomy or cutting down upon the strangled part. Let us suppose that a nurse has been summoned to a case of strangulated hernia in a private house, and then follow the line of procedure that she should follow out under such circumstances. The very fact that her assistance has been needed and asked for may be taken as an indication that operative measures are contemplated. It is in such a case that a competent nurse may be of the very greatest service to her patient, and help to the surgeon. There are three important considerations that should enter the mind of the nurse as she is approaching the residence of the sufferer. The first is that she will be expected to be rapid yet thorough in all her preparations, the second that much of the proceedings necessary for this must be carried out on her own initiative, and the third that the patient should claim her chief attention. I have at times been struck with the fact that a nurse in her zeal to have everything prepared for the advent of the surgeon has unthinkingly, but nevertheless wrongly, neglected the special care and preparation of the patient who is to be submitted to the knife. Emergency operations such as these test to the utmost the capability of the nurse. She is plunged suddenly into a home which is upset from the servants to the master by the knowledge of the fact that one of the household is in a dangerous state, and that an immediate operation may be requisite to save life. The calm, yet decisive manner in which the nurse turns to perform her duties, tends greatly to allay the anxiety and fear which prevails on every hand, and adds much to the ultimate success that is hoped for. On entering the house the nurse should inquire whether the doctor is in attendance, and if possible, secure an interview with him, but if he is not there, she must discover whether he has left any instructions for her. The nurse's first care after this should be her patient. As the case is one of a strangulated hernia, there will be some very characteristic symptoms exhibited by the sufferer. Vomiting will be present, and will have been prolonged, persistent, and usually very exhausting to the patient. Absolute constipation, where neither flatus or fæces are passed, will almost certainly be in evidence. Pain, not only felt in the region of the hernia, but also referred to the navel, will be complained of. It is of no use trying to overcome the vomiting by any means short of the freeing of the bowel from the constriction under which it is placed. No amount of hot or ice-cold water or of effervescing fluid will have the slightest effect on it, and the swallowing of such will only tend to do more harm than good, as it will excite the act of vomiting. The patient under these distressing circumstances is ever ready to appeal for liquid, but it should ever be the duty of the nurse to firmly but kindly decline to satisfy the demand, explaining the reason for the refusal. In like manner it is useless to attempt to give the patient any solid nourishment, for it will only be promptly rejected. It will be evident, therefore, that it is of the utmost importance that relief should be afforded to the imprisoned bowel at the earliest possible opportunity. Moreover, seeing that there is intestinal obstruction, that is to say, the contents of the bowel cannot pass onwards towards the anus, it is of no avail to endeavour to overcome the constipation by the administration of enemata, in fact, if such are given they only aggravate the symptoms. Likewise all purgatives are absolutely negatived. Some relief from the pain may be obtained, in the interval before the operation is undertaken, by the application of hot stupes to the anterior abdominal wall, but this must not interfere with the preparation of the patient, as described below. It is well, therefore, that the nurse should strive to make her charge as comfortable as possible under the circumstances, seeing that the sufferer has had to bear the symptoms with but little assistance before her arrival. A suitable receptacle for the vomited matters should be provided, and water given, so that the mouth may be frequently washed out, without the fluid being swallowed. This will tend to alleviate the continued nausea from the disgusting material that is brought up into the patient's mouth, and at the same time to lessen the thirst, which is often very considerable. Before the nurse turns her attention to the preparation of the apartment, &c., for the operation, she should commence the actual preparation of the patient for the same. The position of the hernial swelling is to be ascertained, remembering that there are three common sites for such protrusions, namely, the inguinal, femoral, and umbilical regions. It is to be borne in mind that the tumour made by the imprisoned bowel may be of very small dimensions, and, therefore, can be easily overlooked. The patient, however, if an adult, is generally able to point to the exact spot. Possibly the doctor in attendance may have ordered the application of an ice-bag to the part, which may be of some use, but it should not be allowed to interfere with the more important preparation of the operation area.

The region of the hernia has to be made as far as possible aseptic before the surgeon makes his incision, and it is therefore desirable that the nurse should seek to obtain this previous to the arrival of the operator, so that no time may be lost. The whole of the parts should be shaved, which proceeding the nurse may have to carry out herself and should be prepared to do so. Immediately after this the area is to be thoroughly scrubbed, so far as the patient will allow it on account of the pain, with soap and water. The soap-suds are next washed off with boiled water, and the parts treated with ether or turpentine to remove the greasy material which always collects on the surface of the skin and is highly septic in nature. Ether is not always at hand, but turpentine is found in most houses. If ether is used it is well to remember that it must not be allowed to come into contact with a naked light, otherwise there is a probability of an explosion. When the excess of the turpentine has been removed, the skin is to be washed over with an antiseptic solution. Perhaps the best to use is one of the biniodide of mercury in the strength of 1 in 1,000. A nurse should always carry some of the tabloids or soloids that are so convenient to prepare antiseptic solutions rapidly. The parts are then covered with an antiseptic dressing, wrung out of the same solution, and so fixed that it is not likely to shift before the operation is begun. It is in this way that the skin may be brought into such a condition that it will be unlikely for suppuration to occur, a process which it is highly desirable to avoid.

I have several times had to thank a nurse for the excellent manner in which she has thus prepared a patient for an emergency operation. If the preparation is put off until the arrival of the surgeon much precious time is lost, and there is less certainty that the parts will be aseptic. In addition to these matters the nurse should see that the patient passes his or her urine before the operation is commenced. It is also very important, especially in cold weather, to make sure that the patient is not unnecessarily exposed to any chilling influences, for he will require all the reserve force that he can command so as to overcome the shock produced by the nipping of the bowel, and the extra amount of the same which may result from the operation. It is therefore advisable that hot-water bottles should be placed near the patient before the operation, the same being removed to the bed into which the sufferer will be carried after the operation, when he is put upon the operating table.

The nurse should now turn her attention to the preparation of the room in which the operation is to be performed. If feasible, this should not be the one in which the patient is, for it is undesirable for all the preparations to be made in his view. Still, it is not always possible to secure the use of another apartment. The first matter that should concern the nurse is the table upon which the patient is to lie during the operation. If it can be found, a table some six feet long, three feet wide, and three feet high will be the most suitable, but this is not often forthcoming. Otherwise the best plan is to use two tables, each some four feet long by two feet six inches to three feet wide. These should be so arranged as to make the form of the letter T, and the pillow for the patient's head is put upon the cross part, while the space on either side of it forms a convenient spot on which to place the towel and basin which the anæsthetist will require if the patient vomits, which he is not unlikely under the circumstances to do. Next to the operation table, the nurse should secure a small table for the surgeon's instruments, and another for his lotion basins. In addition, there should be in the room an ordinary wash-hand stand. It is preferable that as much as possible of the un-needed furniture in the apartment should be removed, as it only tends to obstruct the free movement of the persons that will be present.

The bed upon which the patient is to be nursed after the operation is a point of some importance. A single bed is the best, and it should be provided with a firm, but not too hard, mattress. A feather bed is out of the question for the proper nursing of the patient. The bed should be placed so that it will not interfere with the operator and his assistants during the operation. The position of the operating table is such that the best light may be obtained. A dozen towels are to be provided, and three of these should be placed as soon as possible into a solution of the biniodide of mercury, so that they may be used to surround the operation area. Three good-sized hand basins and three smaller-sized ones, in addition to the one provided for the anæsthetist, should be in readiness. The surgeon will require at least three for his instruments and lotions. A largish receptacle for soiled water and a plentiful supply of boiled water, together with several pints of antiseptic solution, should be made ready. If the nurse happens to have some aseptic absorbent cotton wool she had better place some of it soaking in the antiseptic solution, so that she can easily make swabs of it for the purpose of sponges at the proper time.

It will thus be seen that the nurse has a considerable amount of preparation to undertake, and that in a comparatively short space of time. It is therefore very important that she should have thoroughly mastered what are the essentials of the preparation that she is required to perform. The matters relating to the operation itself and the after treatment of the patient, together with the discussion of the other class of case in which a hernia may be treated by operation, will be deferred until the second lecture.

### Appointments.

Royal Devon and Exeter Hospital, Exeter.—Miss E. Ellen Bath has been appointed Matron. She was trained at St. Thomas's Hospital, London, was for six years sister at the Royal Devon and Exeter Hospital, and since March, 1899, has been assistant matron.

Cork Street Fever Hospital, Dublin.—Miss Maud Weddall has been appointed Assistant Lady Superintendent. She was trained at St. Mary Abbott's Infirmary, Kensington, London, and has since been staff sister at the Infirmary and Children's Hospital, Kidderminster.

### Minor Appointments.

Bethnal Green Infirmary.—Miss F. M. Scott Cavell has been appointed Night Superintendent. She holds a three years' certificate from St. John's House, Norfolk Street, was afterwards staff nurse at St. Thomas's Hospital, charge nurse at the Fountain Fever Hospital, Tooting, and since last May has been ward sister at Poplar and Stepney Sick Asylum.

Walsingham Union Workhouse, Great Snoring, Norfolk.—Miss Annie Morris has been appointed Head Nurse. She was trained at workhouses and the British Lying-in Hospital, and has since held a post at Hambledon Union.

Royal Infirmary, Preston.—Miss Hancock has been appointed Night Sister. She was trained at St. Bartholomew's Hospital, and at the Royal Infirmary, Derby, and has since been sister at the Ear and Throat Hospital, Birmingham, subsequently taking matron's holiday duties.

Gordon Hospital, Vauxhall Bridge Road, London.—Miss Ellen Deadman has been appointed Staff Nurse. She was trained at Wandsworth and Clapham Infirmary, and has since been charge nurse at the same infirmary.

Kidderminster Infirmary and Children's Hospital.—Miss K. Oxley has been appointed Charge Nurse. She was trained at the General Hospital, Birmingham, for three years, and at the Jaffray Hospital for three years.

Shoreditch Infirmary.—Miss Amy Hunt has been appointed Assistant Nurse. She was trained by the Meath Workhouse Attendants' Association at Blenheim House, Kew Gardens.

Woolwich Union Infirmary, Plumstead.—Miss Emily Agnes Hooper has been appointed Head Nurse. She was trained at the Poplar and Stepney Sick Asylum, Bromley, and has been staff nurse at Woolwich Union Infirmary.

### Training in Fever Hospitals.

### SUGGESTIONS BY A CHARGE NURSE.

"SUGGESTIONS by a Sister," appearing in THE HOSPITAL of September 9th, touches part of a very knotty question which, it is rumoured, is shortly to become the subject of a special Norfolk House inquiry, viz., what is the reason of the frequent changes of nursing staff at the various hospitals under the Metropolitan Asylums Board? It is true that a large number of nurses enter under the Board with the intention of only remaining for six months' experience. But the appointments are permanent for good nurses wishing to remain; and in other special hospitals, with less interesting work, it is quite possible to keep charge nurses and sisters for long periods.

It is obvious that posts in general hospitals cannot be obtained by all certificated nurses who prefer hospital work, and a fever hospital presents much of that charm of routine and varied interests to which we have been accustomed, whilst the work, if less interesting, is surely more so than private nursing, with its chances of long engagements and isolated chronic cases. Yet many nurses leave the service of the Board for private work who have really no taste for it.

As "A Sister" writes, the Metropolitan Asylums Board hospitals are certainly to the front as regards comfortable and healthy accommodation for their nurses. The salary is good; so also is the time off duty for recreation. The reasons for resignation usually given are: (1) Inability to work in harmony with the older standing, untrained nurses; (2) the impossibility of obtaining adequate help from assistant nurses who have received no systematic training; (3) the disadvantage of being cut off, on account of the infection, from free social intercourse; and, in those hospitals where charge nurses do night duty, the night work is a great stumbling-block.

The general impression received by a new "charge" is a want of that feeling which in a country would be termed "patriotism" amongst its staff. Perhaps the constant changes and general air of unrest has something to answer for in that respect. The air of patronage which many newly certificated nurses assume towards the hospital to which they come for experience is fatal to a spirit of loyalty, and is, not unnaturally, resented by the older standing nurses; and in those hospitals where there are two charges to a ward, alternating night and day duty, this resentment frequently takes a practical form, especially in those cases where the fellow charge is the one who entered under the old *régime*, and is herself untrained. There are many such remaining under the Board; women who thoroughly understand the fevers and their complications, and who have obtained, besides, much practical experience, during their years of work, of other diseases occurring incidental with the fevers. Less friction would occur, doubtless, if we remembered that their work and special knowledge entitle them to respect, even if we are a little inclined to deride their views on unimportant details; and, since we are now in the majority, surely we can afford the courtesy of omitting to flaunt before them the now requisite certificate, an item which good taste should forbid.

"A Sister's" remarks concerning the average fever assistant nurse show clearly one great difficulty with which a fever charge has to contend. But a seeming lethargy on the part of assistants is often only the reflection of lack of interest on the part of charges. It is so common to hear the exclamation, "Oh; this is very inferior to g neral work! I shall only stay six months," whilst little effort is being made to draw things to a higher level. Enthusiasm, and a willingness to teach on the part of a charge, will often find a response in enthusiasm, and a cheerful readiness on the part of assistants. We should not encourage discontent and contempt for their work; although, by all means, we should point out to them the advantage of general training. Whilst the average assistant nurse is much inferior to the average probationer, there are degrees of competence amongst them, any of which, in a nurse of any standing, is more reliable than a raw probationer's at the commencement of training. All the same, some such arrangement as "A Sister" suggests between the Metropolitan Asylums Board fever hospitals and the various training schools would be an excellent thing.

In regard to night duty, few nurses after a hard training and subsequent staff duty have the requisite freshness to endure a three months' strain of night duty. With a competent night superintendent is it not possible for first assistants to take the responsibility? Many of them have had one or more year's experience in a general hospital. Or at least could not the time be reduced to six weeks? The disadvantage of the infection is not overdrawn, for, although a nurse quickly loses the fear of infection herself, she always knows that her friends are nervous of the possibility of her carrying it outside, and she must feel, to a certain extent, cut off.

These are one or two of the more glaring disadvantages. The fever hospitals are at present much below the level of a well-organised general hospital; but the nurses needed to pull them up are women who will work courageously for their own standard of efficiency, and will not drop to that of the hospital, and selfishly let things slide simply because they only intend to hold office for six months and it is not "worth while to trouble."

---

### SUGGESTIONS BY AN ASSISTANT NURSE.

THERE are always two ways of looking at things. I have been working as an assistant nurse in one of the Metropolitan Asylums Board hospitals for over five years, and I venture to say that the Sister whose suggestions appeared in your paper is mistaken in some points about the assistant nurses in the Board hospitals. It is quite true that there are three classes, viz., those who are refined, intelligent women, and who use their opportunities of acquiring knowledge; those who are capable, but from lack of love for their profession, or indolence, make no effort to become proficient in it; and those who care nothing for the work itself, but who stay because it is convenient to them. Generally, this latter class are those whose mental faculties have not been keenly developed and who have no ambition to excel. On the other hand, may there not be some who earnestly desire a systematic course of training to enable them to become proficient in their work; some who, not being physically strong enough, or from other causes are unable to get into a general hospital for three years' training?

For my part, I sincerely wish the Managers of the Metropolitan Asylums Board would take us as raw probationers and give us a course of training, deferring promotion until certain examinations in fever nursing and other subjects had been passed. We do not wish to pass as "trained nurses," nor are we satisfied with the "little knowledge" we possess, but why do not the "charge" nurses who come from general hospitals lift a little more of the responsibility from the shoulders of an untrained assistant nurse? Why is it that so often we not only have our own work, but the "charge" nurses' too? Is it fair that they should get the credit for what is often due to the plodding perseverance of an assistant nurse? I speak from experience, having seen splendid cases pulled through under the skilful direction of the doctors by an assistant nurse.

I think most assistant nurses under the Metropolitan Asylums Board are not only willing, but grateful to be taught. Those know best who see how essential good nursing is in a fever hospital, and those who have the interest of their patients at heart will do their utmost to attain proficiency in fever nursing, even though they may be only "assistant nurses." "No training necessary" does not mean that no training is desired by us; we do desire it. We thank the authorities of the Metropolitan Asylums Board for their consideration in very many ways for our health and comfort, but we look forward to the day when a systematic course of training with examinations shall be the rule in every Board hospital, so that we shall be no longer despised as possessing only a "little knowledge" whilst we profess a great deal.

### French Hospitals.

By an Occasional Correspondent.

### NURSES OR NUNS?

A point which I am sure will deeply interest English nurses is the question one meets so often with in France, Which is the better?—nursing by lay nurses as a profession or by spiritual sisters as a devotion? In England we see but little of the difference; the nursing profession here is a profession which has laid down a certain standard of training for efficiency, so that any spiritual body taking up nursing would be bound more or less by tacit public opinion to conform to a somewhat similar training. The only experiment that I know of in England at all similar to the nursing in a large number of French hospitals was the nursing of University College Hospital, by the Nursing Sisters of St. John, and apparently that was not found sufficiently satisfactory in all ways, since it was discontinued after a lengthy trial of several years.

It must be remembered, therefore, that there are two distinct methods of hospital nursing in France, *i.e.*, hospitals where the nurses are laymen and laywomen, working under the control of the administrative bureau as personified usually by "*M. le Directeur*," and hospitals, where the nurses are professed nuns, working under the orders of their spiritual chief, "*la superieure*."

At the Hotel Dieu, which is the largest hospital in Paris,* the foundation is a spiritual one. The Hotel Dieu has nurses of both sorts, but so far as I could gather, they do not work side by side as equals, but the professed sisters take the place rather of ward sisters, while the lay nurses take the actual nursing duties under their instruction. In this hospital, therefore, there are practically two schools side by side—a training school for ward sisters and a training school for nurses.

The school for ward sisters consists solely of *religieuses*. When a nun joins the sisterhood she is taken in hand by the senior sisters, and trained by them in all the duties of administration and supervision and routine and control which her future work will require from her. She does not join the lay probationers, either in their work or in their teaching, nor does she have to pass examinations, as they have, for a diploma.

The nursing school, on the other hand, is recruited from the ranks of young women who take it up as a business, just as they take up clerking or typewriting. They are taught by the ward sisters, and also attend classes and obtain a diploma after examination. The difference between this method of training and the one in vogue in England is obvious. Here the sisters are first nurses, and it is from the ranks of the nurses that the ward sisters are taken. The majority of nurses feel that, just as the common soldier has the marshal's bâton in his haversack, so the rawest probationer has the sister's belt in her chatelaine!

At other hospitals—as, for example, that of La Charité† —there are no *religieuses*, and *la surveillante* takes the place of the ward sister in the way I have already described. At other hospitals—as, for example, most of the provincial hospitals—the nursing is practically in the hands of the religious orders.

This is very admirable in many ways. The life of simple devotion, the utter unselfishness, the absence of all those extraneous attractions which injure the work of a large number of lay nurses, the ever-present conception of the sacredness of their calling, the freedom from all the worries that are connected with the hard struggle which many a woman has for life, the definite withdrawal from the great lottery game of marriage: these all combine to make nursing by religious orders beautiful in the extreme.

But there is another side to it. I asked one of the Sisters at the Hotel Dieu what she thought of the English hospital methods, and she told me that not only had she never been out of France, but that she had never been outside the hospital walls since she entered them! It was against the rules of the order!

The same thing struck me at Amiens. There was no interest in, because no knowledge of, the methods of other hospitals. None of that keen competition to have the latest and best of everything, which makes English hospitals so perfect.

A deadly torpor seemed to hang over the spirit of the work. Each sister learned her methods from her preceding sister, and she from the one before, and so on back to the dear old Gamp period. Always deeply honest, always painfully conscientious, but too often hopelessly old-fashioned. Just as it was difficult for the Roman Church to be moved on with the times, and the Reformation became a necessity, so I see that if the nursing were to fall again entirely into the hands of the religious orders there would be good-bye to progress, and the wheels of advancing science would be hopelessly clogged. Taking it all round, therefore, I come back to our own hospital wards with the profound conviction that in respect to nursing, whether as to the social status of nurses, the honour in which the profession is held, the thoroughness of the training, the opportunity for progress, and lastly, but most important, the general well-being of the patients, we in England are well ahead of our neighbours across the Channel.

* The Hotel Dieu stands on the north side of the great square, and is passed unnoticed by the crowds who go by to see Notre Dame on the east side. It accommodates about 850 patients. The old Hotel Dieu, which stood formerly on the south side, is said to have owed its foundation to Clovis II. in about the year 660 A.D.

† Hospital de la Charité, 47, Rue Jacob, between the Boulevard St. Germain and the Seine (516 beds).

### Pass it On.

If you have a kindness shown,
Pass it on;
'Twas not given for you alone,
Pass it on;
Let it travel down the years,
Let it wipe another's tears,
Till in heaven the deed appears:
Pass it on.

### Echoes from the Outside World.

### AN OPEN LETTER TO A HOSPITAL NURSE.

The general opinion is that the reply of the Transvaal Government to Mr. Chamberlain has brought us close to the brink of war. The Colonial Secretary is again in London, and another Cabinet Council is to be held. Is this, one wonders, to be the final meeting before an outbreak of hostilities? Do you know that Lord Salisbury has a strong objection to the time of the Council being made public? It is not that he is an advocate of secret conclaves, but he exceedingly dislikes the manner in which the members of the Cabinet are criticised in some of the papers. On the last occasion, Mr. Balfour's hat, Sir Matthew Ridley's salutation to a colleague, Mr. Walter Long's coat, Mr. Chaplin's smile, and other details were described. It was even suggested that the expression on the faces of Ministers as they came out supplied the clue to the nature of their decision. All this, and much more; rubbish the Prime Minister did not relish. But I am afraid that he is fighting against the inevitable. In these days it is almost impossible to prevent some lynx-eyed outsider, with ears as sharp as his, or her, eyes, from learning that at a certain time on a certain day the Cabinet will assemble in Downing Street; and if once this information is obtained the rest follows as a matter of course. Moreover, if there were not a demand for such trivial particulars, there would surely be no supply. It is often, I confess, a mystery to me that a full and authentic report of the proceedings at each Council is not furnished to the Press—by someone tucked away under the table, or concealed in a corner.

---

It is decidedly a matter for congratulation that the reception of the contingent from L'Association Française pour L'Avancement des Sciénces at Dover, on Saturday, by the British Association should have been of so friendly a character. Now that all has passed off so well, and that the visitors returned home so thoroughly pleased with their entertainment, the officials do not mind admitting that there was much anxiety lest some of the strong partisans of Captain Dreyfus should have thought the arrival of so many French men and women afforded a splendid opportunity to make a hostile demonstration. Had the steamer been welcomed with a storm of hisses it would have placed the British Association, in their capacity as hosts, in a very awkward predicament. The mass meeting in Hyde Park on Sunday also appear to have been managed with skill. The language in which the French Government was besought "to act according to the best traditions of free and generous France by releasing and rehabilitating Captain Dreyfus before it is too late," could not have been more carefully chosen had a reporter been waiting on the spot to telegraph the words straight to Paris, and the meeting must have done at least as much good as harm, which is a great thing to say of a Hyde Park demonstration. Of course, the subsequent news that a pardon had been decided upon and carried into effect by the French Cabinet, is not the result of the meeting, any more than it is that of the attempt to boycott the Paris Exhibition. But there can be no doubt that the manifestation of public opinion in all parts of the civilised world, and in France itself, weighed considerably upon M. Waldeck-Rousseau and his colleagues. If the reports of the ex-captive's health are correct, he has been set at liberty none too soon. Such violent shivering fits that he has had to be wrapped up in blankets and to wear three woollen waistcoats show that the severe shock of the second condemnation, coming after his experiences at Devil's Island, has, at least temporarily, seriously impaired his constitution. His pardon and liberation must have been welcomed not least by the Paris tradesmen, whose business has lately swiftly diminished. One house alone was requested to take the names of thirty-eight customers off their books in three days. The house agents on the Riviera have been kept quite busy receiving telegrams and letters from people who, since the verdict, are declining to go to the South of France for the winter, and here, too, the news cannot fail to have afforded satisfaction. It will be time for Captain Dreyfus to consider the action he shall take in order to secure his full civil rights, and the restoration of his rank in the Army, when he has enjoyed for awhile the novel sensation of freedom and the society of his devoted wife.

---

Lord Kitchener has been more fortunate in the choice of the present which he is sending to Her Majesty than was King Menelik of Abyssinia. It was difficult for even the most lively imagination to picture the Queen bowling along behind a pair of zebras, but the white Arabian ass, which reached Plymouth safely on Tuesday night, is much more likely to be of practical value to the Sovereign. It is to be taken to the Royal Mews at Buckingham Palace till it is known what use the Queen decides to make of the gift, but as she has recently lost a donkey which she used to drive at Windsor, and also because, in common with many others, she is a great admirer of the Sirdar, it is probable that the white donkey will have the honour of being reserved for the personal use of Her Majesty at her Berkshire home.

---

There was a time when the junior clergy waxed rebellious because some of their bishops objected to the cultivation of moustaches. But lately, as you may have observed if you have brothers or cousins, the fashion has been in the other direction, and even young officers in the Army and Navy have taken to shave the upper lip—in some cases where a razor was scarcely required. The Secretary for War, who practises what he preaches, evidently thinks a moustache as desirable among military men as the Archbishop of York considers it undesirable among young deacons and priests. He has issued a circular, which is, of course, tantamount to an order, enjoining upon the officers commanding regiments the importance of reinstating the too often missing appendage. Moustaches, therefore, are likely to be generally worn again, and you will feel additionally interested in the revival when I mention that there is a rumour that Lord Lansdowne's order had its origin in the expression of an opinion on the part of a lady of rank and influence.

---

The title of the new play at Drury Lane is descriptive, more or less, of all the autumn pieces at the same house, for in all Love is invariably triumphant, and, however badly things may go, "Hearts are Trumps" at the last. These spectacular dramas always include realistic illustrations of scenes of everyday life, and the more difficult and apparently impossible they may seem of reproduction upon the stage the more delighted is the audience when it recognises the familiar features of the Derby, or Henley, or the Stock Exchange. This year there is the private view at Burlington House, a children's fête at the Botanical Gardens, a performance at a music hall, and a mountaineering accident on the Alps. The author is Mr. Cecil Raleigh, and the incidents follow each other in such swift succession that there is no time even to think how long the play is, one exciting scene passing so naturally into another that when, soon after emerging from the spacious theatre, the clock strikes twelve, it comes with quite a shock to the playgoer. I mention the time because I know how important punctuality is to nurses, and it is so easy to forget when carried away by enthusiasm. Miss Violet Vanbrugh, as the lady gambler, has a thankless task, and performs it splendidly; Mr. Lionel Brough makes the most of his opportunities, Mr. Cooper Cliffe is a manly lover, and Miss Dora Barton a winsome heroine. Those of you who like to forget for an evening your hospital, patients, routine, and fatigue will find "Hearts are Trumps" an excellent prescription. If you book the seats yourself, go in the afternoon. Then not only can you inspect the exhibition of paintings at the theatre, but you can have tea served you in the picture galleries for nothing. At least, so I am told.

### The Plague in India.

DEPARTURE OF MORE NURSES.—THE TERMS OF ENGAGEMENT.

On Wednesday the following lady nurses left London for Bombay in order to take up their duties as nurses among the plague patients in India: Miss A. M. Cockraft, trained at Addenbrooke's Hospital, Cambridge; Miss May Johnston, trained at the Middlesex Hospital; Miss A. J. Kendall, trained at the Royal South Hants Infirmary; Miss Helena Riddick, Miss Mildred Coulston, Miss Margaret Poole, trained at St. Thomas's Hospital; Miss A. M. Crowdy, trained at St. George's Hospital, and on plague duty in India in 1897; Miss G. M. A. Jessop and Miss Margaret Aukett, trained at the London Hospital.

The following ladies started last week: Miss I. E. B. Hare-Scott, Royal South Hants Hospital; Katherine McIntosh, Leicester, Grimsby, and North-West London Hospitals; M. H. Ross, Royal Southern Hospital, Liverpool; H. G. T. Thompson, Calcutta, Royal South Hants, and St. Mary's, London, Hospitals; A. J. Marshall, trained at St. George's Hospital; H. Herbert, trained at Cumberland Infirmary and at the Eastern Fever Hospital; M. C. Roxburgh, trained at University College Hospital; E. G. Goldney, trained at St. Bartholomew's Hospital; R. L. Massey, trained at London Hospital; A. F. Steer, trained at the Metropolitan, University College, and East London Hospitals; M. L. Rait, of Eastern Hospital, Metropolitan Asylums Board; Marianne Kirkham, trained at Leicester Infirmary; E. S. Platts, trained at Liverpool Northern Hospital; Florence Hodgson, trained at Manchester Royal Infirmary; C. M. Corbett, trained at the Derbyshire Royal Infirmary; C. L. Warrack, Aberdeen Royal Infirmary; Anna Boyd, trained in the City of Dublin Hospital; M. E. Gray, Royal South Hants Infirmary; Ellen Duff, trained at the National Hospital for Paralysed and Epileptic and at the London Hospital; E. E. Bradshaw, trained at St. George's Hospital, and served in India in 1898 on plague duty; and Blanche Crane, trained at Meath Hospital, Ireland.

Prior to their engagement the volunteers had to produce certificates of birth showing that they were between 28 and 35 years of age; to pass an examination by the president of the Medical Board as to fitness for service in India; to produce certificate of at least three years' training at a hospital in which adult males receive medical and surgical treatment, and in which a nursing staff is maintained; also to furnish recommendation from the matron of the hospital at which they have been trained. The engagement is for not less than six months, and each nurse is entitled to a first-class railway ticket and free passage by second saloon P. and O. company's steamer to India and back, though, in the event of resignation within a year, except on account of ill-health, the free return passage from India is forfeited. The remuneration is 175 rupees per month, in addition to free quarters, fuel, lights, and punkah pullers, to commence from date of embarkation; there is an outfit allowance of £10; and travelling expenses from and to residence in England to port of embarkation, with cost of 6 cwt. of baggage, are allowed.

The Under Secretary for India, to whom we are indebted for these details, informs us that at present the full number of nurses needed has been made up, but that names of applicants are recorded in view of possible future needs.

### To Nurses.

In order to increase and vary the interest in the *Mirror*, we invite contributions from any of our readers in the form of either an article, a paragraph, or information, and will pay a minimum of 5s. for each contribution. All rejected manuscripts are returned in due course, and all payments for manuscripts used are made at the beginning of each quarter, *i.e.*, January 1st, April 1st, July 1st, and October 1st.

### Lines to a "Mere Pro."

(A Rejoinder, *Vide* September 9th, 1899.)

Probationer, pert probationer,
Will nothing worry *you*?
Not even if your carelessness
Should kill a man or two?

You give an "intestinal case"
A pint of milk to drink,
And when he nearly breathes his last,
You say, "I didn't think!"

You take a "mitral" to the bath,
You give a "typhoid" meat;
You put hot bottles next his skin
And blister both his feet.

When doctors, clerks, and class come round,
With shame you ought to sink;
Their patient, whom you've just washed "clean?"
Has feet as black as ink.

The "specimens" you're told to save,
You always throw away;
But "Sister" 'tis, who has to face
The "Honor'ry" next day.

You break thermometers galore,
You quite forget to dust;
You let the instruments boil dry,
And leave them so, to rust.

You're shown your work a dozen times,
"Thou shalt," and "Thou shalt not";
But still "I didn't know" you cry,
Or else "I quite forgot."

No wonder "Sister's" face is long,
Her temper sometimes "short";
Your blunders may be someone's death,
To you, perhaps, they're sport.

She may not speak in honeyed words;
Remember all the same,
When things go wrong, 'tis not the "pro"
'Tis "Sister" bears the blame.

A Hospital Sister.

### Raising the Wind at Penrith.

A most successful bicycle gymkhana was held a short time ago at Penrith in aid of the cottage hospital recently erected there, and shortly to be opened. The promoters and supporters were charmed with everything.

The day was delightful, and the spectators crowded in from all parts. The programme was of a most attractive nature, and included a very pretty and effective musical ride given by sixteen ladies and gentlemen, while last, but not least, the sum handed over to the charity was unexpectedly large. A considerable number of dainty and handsome prizes were afterwards distributed by the Countess of Lonsdale, who took a warm interest in the proceedings throughout.

The receipts were as follows:—Gate, £70 10s. 6d.; grand stand, £19 0s. 6d.; tea tent, £12 14s. 3d.; programmes, £3 19s. 2d.; collection in boxes, £2 6s. 1d.; open-air dance in the evening, £4 5s.; total, £112 15s. 6d. The committee were able to hand over to the treasurer of the hospital £80, which sum is to be added to the Endowment Fund.

### The Nurses' Bookshelf.

[We invite Correspondence, Criticism, Enquiries, and Notes on Books likely to interest Women and Nurses. Address, Editor, The Hospital, (Nurses' Book World), 28 & 29, Southampton Street, Strand, London, W.C.]

Nursing: Its Theory and Practice; Being a Complete Text-book of Medical, Surgical, and Monthly Nursing. By Percy G. Lewis, M.D., M.R.C.S., L.S.A. Thirteenth Thousand. Enlarged and Revised throughout. (London: The Scientific Press. 1899. Price 3s. 6d.)

A Handbook for Nurses. By J. K. Watson, M.D. (London: The Scientific Press. 1899. Price 5s.)

We have before us two books on nursing which, good as they both are, differ much in scope and character, as indeed nurses also do both in character and in ambition. One of these works is an old friend, "Nursing, its Theory and Practice," by Dr. Lewis, having now reached its thirteenth thousand, a signal proof that it has provided a large number of readers with the sort of information which they required. This edition has been enlarged and revised throughout, and this has been done so thoroughly that without the general arrangement of the work being materially altered we find several new subjects dealt with, chapters having been added, as is stated in the preface, "on Mental Nursing, Mechanical Therapeutics, Massage, Schott, Tallerman, and Weir-Mitchell Treatments." There can be no doubt that in their effort to make their book a kind of "Inquire within on everything relating to nursing," the author has been wonderfully successful, and if, as is the fact, the information given does not in some parts go very deep, it enters quite as deeply into the various subjects discussed as most nurses require, and quite as deeply as the size of the book admits, for there is no waste space and no useless padding. So far as it goes it is an admirable nurse's handbook, dealing with anatomy and physiology, the various diseases and accidents met with, and the various lines of treatment adopted, but always dealing with them in such a way as to lead up more especially to the nurse's duties in each case. It is a book which may be confidently recommended, and its success has proved its utility.

We now turn to the "Handbook for Nurses," by Dr. Watson. This is a very different book, and the probationer who takes it up in the expectation of learning how to nurse will probably be disappointed, for of nursing, *per se*, there is but little, all the temperature-taking, the bed-making, the sheet-changing, and other "tricks of the trade" being squeezed into one chapter. What then is the book about? It really is a handbook, not of nursing, but, as it says on its title-page, "for nurses"; a handbook dealing not with nursing itself so much as with all the things a nurse wants to know if she is to take an intelligent interest in her work —anatomy, physiology, medicine, surgery, and obstetrics. We know that there are plenty of people who will say that this cannot be necessary for a nurse. Nor is it. There are plenty of good nurses who, being blessed with those two admirable virtues, obedience and common sense, get on capitally with but little "knowledge" in the modern sense. But if intelligent and well-educated women are to devote their lives to nursing, not merely as an expression of religious zeal, but as their main aim in life, it does not seem entirely unreasonable that they should know something of the nature of the diseases from which those are suffering whom they nurse, and of the aims and objects of the treatment the carrying out of which is one of their main duties Indeed, we know well enough how constant and urgent are the efforts of some of the best probationers to get hold of medical and surgical books in the hope of digging out of these ponderous volumes the information they want as the key to what they see going on around. "Curiosity, thy name is woman," has often been said; but it need not be said in a tone of blame. A desire to know what it is all about must possess any healthy-minded woman who finds herself plunged into the mysteries of the treatment of disease, and in this book she has the key. As we said before, the book is not necessary, but it is just the sort of short handbook to medical subjects which nurses want.

### The Brighter Side of Workhouse Infirmary Nursing.

### AN OUTING FOR INMATES.

The Superintendent Nurse of a provincial Workhouse Infirmary sends us the following:—

"I have been thinking that there are a few of my colleagues, not Poor Law nurses, who might care to hear of one of the many pleasures that come in our daily routine of duty. I have in mind just now a garden party given yearly by some kind ladies, to which fifteen of the most infirm inmates were invited. The difficulty was to get the old women to consent to go. They made such excuses as 'I am too much trouble,' or 'I could not possibly give so much trouble.' However, when I assured them that being helpless, blind, and otherwise infirm must not be an excuse for refusing such a kind invitation, they promised to 'think' about it. I was specially asked to go, so nurse dressed them while I made my own preparations. Two of the party were wards-women, not even able-bodied, but they were most useful in helping to assist with the old ladies. The brake arrived at two p.m. It was a delightfully warm day. We managed to get the women up into the brake, and when we heard the programme was to drive first it gave great delight. Some had not been out for months, others for years. The drive had been carefully planned, so that we should see the nicest part of the suburbs. It was delightful, under avenues of trees, and past brilliant gardens, and then down the promenade. It gave special pleasure to see on one side gay shops and gaily dressed ladies; on the other side, beautifully kept gardens. After an hour's drive we arrived at the end of our journey. We were received by the ladies, anxious to assist us to alight. All were safely helped in to the garden, and there we were very warmly greeted. A few special friends from the parish church's mothers' meeting were invited to join us. This was a yearly arrangement, so it seemed like meeting old friends. The hostess had sons, quite young men, who amused the old ladies, and were so kindly and courteous that it did one's heart good to look at them. The garden was sheltered and shady, and very, very bright with flowers. There were several tables, all prettily laid out with plants, flowers, nicely-arranged cakes, &c. By each plate was a buttonhole of roses, jessamines, and fern. The young gentlemen helped to pin in the buttonholes, and it caused such fun that one could imagine all the party young again. The tea was daintily served and much enjoyed. Afterwards the young ladies assisted their guests to see the garden and the poultry. In the meantime seats were arranged, and the remains of tea removed, so they might enjoy a gramaphone entertainment. This gave great pleasure —they could not understand where the voices came from! some very quaint remarks were passed. After the entertainment refreshments were handed round, and then the brake arrived (quarter-past six p.m.), so we hastened to take our seats. On leaving, each guest was given a packet of tea, sugar, and sweets. The remaining cake, jam, &c., was packed up for us to take back to the bed-ridden patients. When all were safely seated, after bidding good-bye, the old ladies gave their heartiest hurrah! The coachman was requested to take us another hour's drive and by another route. It was delightful, and we arrived home at half-past seven p.m., having had a most delightful time. Poor old ladies, they had enough to talk of for many days. I am sure they did not enjoy themselves a bit more than the nurse."

### Everybody's Opinion.

[Correspondence on all subjects is invited, but we cannot in any way be responsible for the opinions expressed by our correspondents. No communication can be entertained if the name and address of the correspondent is not given, as a guarantee of good faith but not necessarily for publication, or unless one side of the paper only is written on.]

### IODINE STAINS.

"SISTER" writes: "It may interest some of the readers of THE HOSPITAL, who are not already cognisant of the fact, that the stains of iodine on linen, glass, camel's hair brushes, &c., may be at once removed by immersing the same for a few minutes in carbolic acid lotion, 1 in 20. Similar stains on the fingers may also be removed by rubbing them gently with gauze soaked in the same solution. As the remedy is within the reach of every nurse in these antiseptic days, it may prove of value to some."

### NURSES' DRESS.

"M." writes: I am interested to see the letters about this question by "M. C." and "A Private Nurse." I still venture to think that a little change and amendment is wanted even in the dress of some of those who are not sham but real nurses. So often I am struck by the untidiness, and, if I may coin a word, the fly-away-ness of some nurses' dresses. Floating cloaks, floating veils, and a large expanse of apron really are not necessary portions of a nurse's out-door attire. I do not altogether agree with "A Private Nurse" as to nurses not wearing uniform. In many parts of big towns uniform is a very great protection, and a nurse can often go fearlessly into places where even a policeman cannot venture. But certainly in other places there is no reason why a nurse should not go out in ordinary clothes.

"M. C." writes: In common with many trained nurses of my acquaintance I cannot altogether agree with "A Private Nurse" in wishing to discard out-door uniform altogether, because it seems to me that the purpose for which the uniform was primarily designed was like that of the Sisters of Charity —to denote the profession of the wearer; and if trained nurses, disgusted by the use of bonnet and cloak by women who have no right to wear them, were to unanimously adopt private dress out of doors, to what deeper depths of degradation would not the already too-much-abused uniform sink? Its use would simply, in course of time, imply to the general public that the wearers were merely ignorant and untrained women, if not actually disreputable characters, and that respect which, I am thankful to say, is still commanded by the nurse's uniform, despite its numerous abuses, would be turned into contempt. As to parading one's self, surely a Sister of Mercy is none the less modest, womanly, and self-effacing because she has adopted a distinctive dress; and, in my opinion, out-door uniform ought similarly to be the badge of the self-sacrificing, gentle, and womanly lives led by its wearers; not as, at present, it too often is, merely the insignia of feminine vanity, conceit, and foolish affectation, if not worse.

### "ONE OF THE FAMILY."

"VERA" writes: Has it ever struck those nurses who write on the question of having breakfast in their bedrooms that possibly it may not be either convenient or feasible to treat a nurse as one of the family? In a great many households it is not at all easy to arrange that a nurse should have her meals in the dining-room and sit in the drawing-room when off duty. For instance, grown-up sons living at home do not always like it, or the father or mother of the household prefer not to have a stranger at meals, and, so to speak, constantly in the midst of family things. You see, "the family" has to be considered as well as the nurse; and, after all, a nurse must remember she is a stranger in the household, and members of it do not perhaps care to have her a great deal with them. Nurses are not sufficiently reasonable, I think, about this. No personal affront is intended when people are obliged to arrange that a nurse should have meals separately, and a nurse worthy of the name tries to adapt herself to the exigencies of the house in which she is, and does not look out for affronts. Of course, it is quite easy to ask not to have your meals in a bedroom, and usually it can be arranged to give them you either in the dining-room after the family meal is finished, or in some other room. But it does seem to me a little petty to make an undue fuss over the question of where you have your meals. And may I venture to say that I am not surprised that people do not care to make nurse one of the family, when I hear the way in which nurses talk of cases and families after they have left them? It is, indeed, no wonder that families keep nurses at arm's length when one hears how some nurses talk of families.

"AN OLD NURSE, RATHER FOND OF MENIAL THINGS," writes: The correspondence on this subject is not a little puzzling. Does the "Matron, Patient, Nurse," think the grand profession injured by the nurse doing work a secretary or governess might scorn? The long (?) and arduous training, the cultivated touch (?) is doubtless likely to impress some folks with sublime ideas of importance; but will the standard be lowered by the nurse's kindly accommodation of herself to existing conditions? Private nurses must take the rough with the smooth. Where there is only one poor little maidservant kept the nurse won't lower the profession by "turning to," even with broom and dish-cloth, and where the family is able to keep three or four servants the nurse will find, if she is of the right sort, that the good sense of the family will know how to treat her with courtesy and respect, so that her brain power and cultivated touch may not be thrown away. Many an earnest country doctor is not afraid (where the need arises) to make up fires, and attend to many menial offices in a cottage home; and some of our noblest and most refined nurses are amongst those who do their best by their surroundings, and talk the least of "the profession."

"BETH" writes: I think the discussion, "Breakfast in the Bedroom," or "One of the Family" very amusing, as at the present time I am nursing a case and am taking my breakfast in my patient's room with her every morning, and we find it very pleasant indeed. I cannot say I should always like to do so, or at all cases. I have never been asked to have my meals in the kitchen with the servants. When I am engaged to take a case people sometimes inquire where I like my meals. I say either with the family or they can be sent up to a sitting-room or my dressing-room I have usually been treated very nicely, but of course there are some exceptions, and if everything is not quite pleasant there is the consolation that it is not for always, and one only lives one day at a time. I have been private nursing several years, but find I cannot pick and choose my cases. I am generally treated as "one of the family," but it depends a great deal upon the class of the patients, and in some cases I would rather not be treated so. I think, after all, it is of very little consequence where we have our meals, as a true nurse's chief thought is for her patient, and my experience is that where the patient's friends see a nurse is doing her level best to get the patient well they will study her and her comfort, especially when they realise that she thinks very little of herself and her own dignity.

### HOURS OF REST FOR PRIVATE NURSES.

"C. N. S." writes: I should like to know what hours of rest private nurses consider the most convenient. When working two together I have always changed at eleven o'clock, the day nurse remaining to help the night nurse with anything she might need, and *vice versâ*. Coming on duty at eleven p.m. seems to make the night so very much shorter, and gives both nurses a chance of getting out for a little fresh air in the morning and of having eight hours in bed. Then usually the doctor has arrived about eleven a.m., and both nurses can explain their reports and receive orders. I find the most difficult thing to arrange is when working alone, and doing the night work. Usually people like a nurse to go to bed after dinner. This meal is frequently at half-past one, which means it is half-past two or a quarter to three before she gets into bed, then at nine p.m. she is called, and by the time she has dressed, had a meal, heard all that has happened in her temporary absence, put the patient ready for the night and seen that all needful is at hand, it is nearly eleven p.m., and a nurse (at least, speaking personally) does not care to keep a daughter, or whoever has been in charge, till eleven p.m. every night.

### For Reading to the Sick.

"There is one Body and one Spirit, even as ye are called in one hope of your calling; one Lord, one Faith, one Baptism, one God and Father of all, Who is above all, and through all, and in you all."—*Ephes.* iv. 4-6.

Elect from every nation
Yet one o'er all the earth :—
Her charter of salvation
One Lord, one Faith, one Birth!
One Holy Name she blesses,
Partakes one Holy Food,
And to one hope she presses
With every grace endued. —*Stone.*

It is the Christian tradition that God has organised His Church as the sphere in which mankind may, in this life, be received into a state of grace. It is held that in the Church Jesus Christ, through the agency of His Spirit, raises men out of their fallen state, and places them in the state of grace, and thus fits them for the life of glory hereafter. The Church on earth is thus set forth as the highway of grace, the sure and certain road along which mankind may pass to glory. It is held that in the Church the work of redemption and restoration is carried on by the Holy Spirit uniting men to Jesus Christ, the redeemer and the restorer of humanity.

Jesus Christ came to restore man to fellowship with God as a race. By the Incarnation Christ entered into the ranks of human nature, and assumed a real relationship to the whole of humanity. As a race, mankind had, in the fall of the first Adam, lost fellowship with God. As a race, mankind in Jesus, the second Adam, may be restored to fellowship with God. "For as in Adam all die, so also in Christ shall all be made alive." Christ died to take away "the sin of the world," the sin of humanity; and He rose to life, and sent down His Spirit to renew humanity as a whole.

Thus, there comes home to the Christian consciousness the idea of the Church as a world-wide and enduring society, in which reside the spiritual forces of redemption and restoration which flow from Jesus Christ. These spiritual forces we know as grace, "the power that worketh in us." Grace is the power whereby Jesus Christ works upon the hearts, the minds, and the wills of mankind.

It is the teaching of Christ that His grace should be assured to mankind in the Church, as the sphere of grace, by means of outward and visible signs, or sacraments. He declared that He would bestow a new birth upon the soul by the sacrament of water and the Spirit. It is the teaching of Christ that by the Spirit's operation the new life of Christ is planted in the soul in Baptism, and that this new life is supported and increased by a real though spiritual feeding upon Himself in the Communion of His Body and Blood. —*Vernon Staley.*

Crowns and thrones may perish,
Kingdoms rise and wane,
But the Church of Jesus
Constant will remain;
Gates of hell can never
'Gainst that Church prevail;
We have Christ's own promise,
And that cannot fail. —*Baring Gould.*

### NURSES AT TRACHEOTOMY DRILL.

Our correspondent writes: "I regret to say that in reporting the above I made an error through misapprehension. Although the lecture-room demonstrations are open to the whole of the nursing staff, the actual drill, as described, is only practised by the assistant nurses in Dr. G.'s own diphtheria wards."

### Notes and Queries.

**The contents of the Editor's Letter-box have now reached such unwieldy proportions that it has become necessary to establish a hard and fast rule regarding Answers to Correspondents. In future, all questions requiring replies will continue to be answered in this column without any fee. If an answer is required by letter, a fee of half-a-crown must be enclosed with the note containing the enquiry. We are always pleased to help our numerous correspondents to the fullest extent, and we can trust them to sympathise in the overwhelming amount of writing which makes the new rules a necessity.**

**Every communication must be accompanied by the writer's name and address, otherwise it will receive no attention.**

*A Nurses' Association.*

(222) In forming an association of trained nurses is registration necessary? What are its advantages, and where should one apply for definite information as to the steps to be taken?—*Nurse.*

Your question is somewhat vague, and gives no indication of the sort of "association" which it is proposed to form. We may point out, however, that if it is a private venture of your own there would seem to be no special object in incurring the expense of incorporation, which would amount to from £50 to £100. If a public association of nurses is contemplated, worked by a committee, then the need for incorporation would not arise until it is established and its plan of work definitely settled by experience. When that time arrives the services of a solicitor would be required to prepare the necessary papers for incorporating the association under the Board of Trade should such a step be decided upon.

*Bicycles.*

(223) Could you tell a nurse, living near Charing Cross Station, where she could keep her bicycle, so as to get at it easily when off duty in the evening? There is no accommodation for it in her lodgings, but she would be willing to pay a trifle for its keep.—*M. N. J.*

Most of the bicycle shops offer facilities for stabling cycles. Make inquiries as to terms at those most convenient of access.

*Paris.*

(224) Could you give me addresses of institutes for English nurses in Paris, or where I could obtain information of such? I want to nurse in Paris. I speak French fluently.—*Nurse Elenor.*

The Hollond Institute (London headquarters, 1, Tavistock Chambers, Bloomsbury, W.C.) has a branch in Paris, the directress of which would be able to furnish you with reliable information.

*Family Medicine.*

(225) Could you kindly tell me of a medical work suitable for a family about to emigrate; also one for ladies, other than Chevasse, for the same purpose?—*K. O.*

The following would probably suit you: (1) "A Manual of Family Medicine and Hygiene for India," by Sir W. J. Moore, 12s., publishers, Messrs. J. and A. Churchill; (2) "Hints to Mothers on the Management of their Health during the Period of Pregnancy," by Dr. Thomas Bull, 1s. 6d., publishers, Messrs. Longmans and Co.

*Cannes.*

(226) I should be so much obliged if you could give me the address of Mrs. Dan Norris, or tell me in which way I could obtain it? Mrs. Norris has a villa in Cannes for the winter season, and I believe takes out English nurses with her.—*Army.*

A letter addressed to Mrs. Dan Norris, Cannes, should she be a person of importance, will probably reach her.

*Crumpsall.*

(227) Kindly tell me if the Crumpsall Infirmary is a recognised training school for nurses?—*Inquirer.*

Crumpsall Workhouse Infirmary is an important school, and the training is recognised by the Local Government Board.

*Niger.*

(228) Will you kindly tell me to whom application should be made for nurses wishing to nurse on the West African coast (Niger)?—*Nurse J.*

The nurses now with the troops on the Niger were sent out by the Colonial Office, Downing Street. If you wrote to the Secretary you would find out if more volunteers were required. The Colonial Nursing Association, The Imperial Institute, W., would also be likely to find you work on this coast—that is always provided you were suitable for it.

*Army Nursing Staff.*

"*E. S. B.*" is asked to refer to our rules at the head of this column.

*Medical Nursing Lectures.*

"*B. K. T.*" should refer to the review of Dr. Watson's work in the "Nurses' Bookshelf" this week. "Matron" suggests that she might find of use "Notes on Medical Nursing," by the late Jas. Anderson, M.D., &c., published by Lewis, price 2s. 6d.; also "Lectures on Medicine to Nurses," by Herbert Cuff, published by J. and A. Churchill, price 3s. 6d.

### ANSWERS REQUESTED.

*Irish Prisons and Schools.*

(229) Would anyone kindly let a hospital nurse know what prisons in Ireland employ trained nurses to take care of the sick; also to whom she should apply for general information as to salary, accommodation, and hours on duty? Are trained nurses employed (as in England) in the infirmaries attached to large boarding schools or colleges in Ireland. If so, what is the usual salary, and what are the duties required.—*I. H. N.*

Considerable interest attaches to both these questions, as they relate to comparatively new openings for trained nurses. Will our readers, therefore, who have personal experience kindly give full particulars?

### Travel Notes.

BY OUR TRAVELLING CORRESPONDENT.

### XXXV.—COBLENTZ.

WHEN last we talked of the Rhine we made our starting place to enjoy its beauties at Konig's Winter, now we will go higher up and learn what is to be seen from Coblentz.

### COST OF THE JOURNEY.

The cheapest way of all is by the Netherlands Company's steamer *viâ* Rotterdam and the Rhine, £1 19s. 5d. first-class return, and £1 7s. 2d. second-class return. This is a most agreeable way of travelling, the only disadvantage being that you spend too long on the journey if your holiday is limited. It takes from three to three and a half days from Rotterdam to Coblentz, but it is a most restful and agreeable journey. The next cheapest route is *viâ* Harwich and Rotterdam, Cologne and Bonn, by rail, second return, £2 12s. 11d. You can change to the boat if you like at Cologne, but it all adds to the trouble and expense, and you will have sufficient chances to see the river scenery during your stay. Have your luggage booked through to Coblentz, and you will have no trouble at Cologne, where you can see the cathedral comfortably in the two and a half hours between the trains, and have luncheon if you like in one of the many restaurants in the Dom Platz. You will arrive at Coblentz at 5.12 p.m.

### ACCOMMODATION.

I like the position of the Hôtel Giant best. It is so pleasantly placed on the river and close to the steamboat pier; but it is not very cheap. Pension from 7 marks, which is, in fact, 7 shillings. The Central Hotel will take you for 5½ marks, and the Hôtel de Trêves for 5 marks. There are some pensions, but not cheaper than the hotels, and I like the freedom of hotel life better myself.

### ATTRACTIONS OF THE TOWN.

In Coblentz itself there is not much, either in art or architecture, to detain the admiring tourist; but its situation is its attraction, standing at the confluence of the Rhine and the Moselle; its position, sheltered by the frowning fortress of Ehrenbreitstein, is unique. The church of St. Castor, dating from the twelfth century, though of a much older foundation, is fine; it is an interesting church, and close by stands what is called the Castor-Brunnen, an obelisk somewhat vain-gloriously set up by the French in memory of the campaign of 1812. The irony of fate caused the Russian military commander of Coblentz in 1814 to add these words: "Vu et approvê par nous, Commandant Russe de la Ville de Coblence, 1814." The palace will not detain you long; it is painfully restored, and looks very new. Here is held the English service in a room close to the palace chapel. The glory of Coblentz is the Rhine Promenade, on which the rank and fashion of the town takes the air, and where every afternoon the military bands discourse sweet music in the Trinkhalle, whilst the audience listens, flirts, and drinks its beer or coffee.

### EHRENBREITSTEIN.

Visitors, with few exceptions, are admitted to this vast fortress, which bears the name of the Gibraltar of the Rhine. It is 385 feet above the river, and on a hot day it is a somewhat painful pilgrimage to the top walking between walls which afford one very little access to the air and on which the sun strikes down and cooks one as in an oven. But it is an expedition not to be missed; a smart sergeant will take you round, and if you understand German his explanations are most interesting.

### THE ENVIRONS OF COBLENTZ.

If you are cyclists you will not probably trouble the steamers or railways much, but there they are, making excursions up and down the Rhine on both sides of the river wonderfully easy and cheap. The Castle of Stolzenfels is quite near to Coblentz; train to Capellen, and a leisurely ascent of 300 feet up to the castle. Oberlahnstein, on the opposite side of the river, with its Schloss, Martinsburg, and Braubach commanded by Schloss Marksburg, makes a pleasant excursion. On the same side you will see Schloss Liebeneck above Osterspai, considered by artists one of the most picturesque of the Rhine castles. Fifteen miles from Coblentz is Boppard, once charmingly old-fashioned, but alas! the hand of the restorer has been heavy on it. However, its lovely position cannot be spoilt.

### BOPPARD AS AN ALTERNATIVE CENTRE.

If you prefer a quiet town to a noisy one you might like to make Boppard your resting-place, and it is quite as convenient as Coblentz, except for exploring the Moselle country, which, indeed, should be undertaken on a separate holiday. If you stay here, go to the Hôtel Lange, pension from 4 marks. The church is worth seeing (twelfth century work), also the Carmelite church and the castle of the Archbishops of Trêves. There are lovely walks around the Mühlbad. A small wooded valley leads up from it, and from the Alte Burg a wonderful view of the Rhine called "the four lakes" is to be seen. Beyond Boppard, on the opposite side, Steirenberg and Liebenstein, twin castles called "the brothers," rise above Bornhofen. Then comes St. Goar, with the Castle of Rheinfels above, and then very soon the Lurlei rocks. There is hardly half a mile anywhere between Coblentz and Mainz that does not teem with legendary and historical interest. At Cavb observe the singular building in mid-stream called the Pfalz; the only entrance is by a ladder. Here the Electors were wont to retire for safety when their subjects rebelled against their iron rule. Then comes Bacherach, of which I should like to speak more at length another day, for it is a charming centre for excursions. Above it is Schloss Stahleck, the ruins of which only remain. Further on you will pass Sooneck and Falkenburg, and on the same side the interesting Clemenscapelle. There is a curious arrangement here for throwing a light up the river for night navigation. The Mouse Tower, with its old legend of Archbishop Hatto, was doubtless also a watch tower; it is situated in the middle of the stream. From Coblentz to Bingen is quite an easy day's excursion, either by steamer or rail, and on a cycle seems nothing, the road is so good. The country from Bingen to Mainz is not so striking, but I shall hope to speak of it some day. If you take the train some morning to Bingen quite early you will be able to walk to the Rochus capelle, a chapel on the east side of the Rochus berg. The chapel is not of great interest, but the walk is lovely, especially if continued by the Scharlachtropf. The wooded heights of the Rochus berg afford numerous lonely walks and views up and down the Rhine.

### TRAVEL NOTES AND QUERIES.

ROME (Speranza).—I do not think you could see Rome with any comfort in less than a month, though a fortnight is better than nothing if money is of consequence. You are choosing the most expensive route. The cheapest way is by Dieppe, Modene and Genoa. Second class £6 6s. 2d., and third class £3 17s. 11d. If you do not mind the journey being tedious third class is quite bearable.

OBERAMMERGAU (Stevenage).—Arrangements are not yet completed by the railway companies, &c., for the Passion Play. Dr. Lunn is organising a party, the cost of which will be about 25 guineas. There is immense competition for seats, and therefore it is desirable to book your seats early. The performances commence on May 24th, and are held at intervals of a few days till September 31st. Other parties will be organised by Messrs. Gaze and Messrs. Cook, of which I will give you notice. Undoubtedly it is cheaper to go with one of these parties, and probably more comfortable. If you prefer to go independently write to me again, and I will tell you how to manage. The journey (if taken alone) is £5 9s. 11d. second class return to Munich, and approximately the distance beyond both ways will cost you another £2. Probably these prices may be a little lowered by the companies.

